# Expression and Regulation of Cav3.2 T-Type Calcium Channels during Inflammatory Hyperalgesia in Mouse Dorsal Root Ganglion Neurons

**DOI:** 10.1371/journal.pone.0127572

**Published:** 2015-05-14

**Authors:** Masaya Watanabe, Takashi Ueda, Yasuhiro Shibata, Natsuko Kumamoto, Shoichi Shimada, Shinya Ugawa

**Affiliations:** 1 Department of Anatomy and Neuroscience, Graduate School of Medical Sciences, Nagoya City University, Nagoya, Japan; 2 Department of Neuroscience and Cell Biology, Osaka University Graduate School of Medicine, Suita, Japan; Boston Children’s Hospital and Harvard Medical School, UNITED STATES

## Abstract

The Cav3.2 isoform of the T-type calcium channel is expressed in primary sensory neurons of the dorsal root ganglion (DRG), and these channels contribute to nociceptive and neuropathic pain in rats. However, there are conflicting reports on the roles of these channels in pain processing in rats and mice. In addition, the function of T-type channels in persistent inflammatory hyperalgesia is poorly understood. We performed behavioral and comprehensive histochemical analyses to characterize Cav3.2-expressing DRG neurons and examined the regulation of T-type channels in DRGs from C57BL/6 mice with carrageenan-induced inflammatory hyperalgesia. We show that approximately 20% of mouse DRG neurons express Cav3.2 mRNA and protein. The size of the majority of Cav3.2-positive DRG neurons (69 ± 8%) ranged from 300 to 700 μm2 in cross-sectional area and 20 to 30 μm in estimated diameter. These channels co-localized with either neurofilament-H (NF-H) or peripherin. The peripherin-positive cells also overlapped with neurons that were positive for isolectin B4 (IB4) and calcitonin gene-related peptide (CGRP) but were distinct from transient receptor potential vanilloid 1 (TRPV1)-positive neurons during normal mouse states. In mice with carrageenan-induced inflammatory hyperalgesia, Cav3.2 channels, but not Cav3.1 or Cav3.3 channels, were upregulated in ipsilateral DRG neurons during the sub-acute phase. The increased Cav3.2 expression partially resulted from an increased number of Cav3.2-immunoreactive neurons; this increase in number was particularly significant for TRPV1-positive neurons. Finally, preceding and periodic intraplantar treatment with the T-type calcium channel blockers mibefradil and NNC 55-0396 markedly reduced and reversed mechanical hyperalgesia during the acute and sub-acute phases, respectively, in mice. These data suggest that Cav3.2 T-type channels participate in the development of inflammatory hyperalgesia, and this channel might play an even greater role in the sub-acute phase of inflammatory pain due to increased co-localization with TRPV1 receptors compared with that in the normal state.

## Introduction

T-type (low-voltage activated [LVA]) calcium channels were first reported in peripheral sensory neurons of the dorsal root ganglia (DRG) [1]. There are three types of the α1 subunit in T-type channels, α1G (Cav3.1), α1H (Cav3.2) and α1I (Cav3.3) [2], which likely contribute to the observed heterogeneity of T-type calcium currents in native cells. A subpopulation of high-voltage activated (HVA) calcium channels are activated by strong membrane depolarizations, and these channels are important for shaping action potentials and regulating transmitter release. However, T-type channels become activated after small depolarizations of the neuronal membrane (i.e., near resting membrane potential), and these channels are therefore thought to regulate cell excitability [3].

Recent *in vitro* electrophysiological and *in vivo* pharmacological, molecular, and genetic studies have revealed that T-type channels containing the Cav3.2 isoform of the α1 subunit play pivotal roles in the acute nociceptive processing induced by reducing agents, including dithiothreitol (DTT), the endogenous amino acid L-cysteine, nitrous oxide (N_2_O or laughing gas) [4] and hydrogen sulfide (H_2_S), in rats [5]. Cav3.2 channels also contribute to the chronic pain symptoms that are associated with peripheral axonal injury in rats [6–10]. However, there are conflicting reports on the function of Cav3.2 or T-type currents within DRG neurons in rats and mice. In rats, high densities of T-type currents were observed in capsaicin- and isolectin B4 (IB4)-positive nociceptors in dissociated DRG neurons [11]. By contrast, Cav3.2 channels were enriched in capsaicin-insensitive low-threshold mechanoreceptors (i.e., D-hair cells) in mice [12,13,14]. In addition, selective silencing of Cav3.2 improved the paw withdrawal thresholds for tactile stimuli in rats following chronic constriction injury (CCI) [6], and blockage of T-type channels with mibefradil significantly normalized painful behaviors and the hyperexcitability of neuronal firing in a spinal nerve ligation (SNL) rat model [15]. However, the behavioral responses in Cav3.2 knockout mice were not significantly different from those of wild-type animals with respect to SNL-induced neuropathic pain [16]. The roles of Cav3.2 in hyperalgesia and allodynia have largely been studied in rats with neuropathic pain and diabetic neuropathies [17–20], although how T-type channels are involved in acute and sub-acute hyperalgesia induced by inflammatory stimuli is poorly understood.

The present study utilized a typical mouse model of inflammatory pain using carrageenan injections into the hindpaw. We examined the expression and upregulation of T-type channels in DRGs from mice treated with or without carrageenan, characterized the properties of DRG neurons that upregulated Cav3.2 channels during hyperalgesia, and investigated whether multiple T-type channel blockers, including mibefradil and NNC 55–0396, could relieve carrageenan-evoked inflammatory mechanical hyperalgesia.

## Materials and Methods

### Animal use and care

All experimental protocols were approved by The Nagoya City University of Medical Animal Care Committee (Permit Number: H25M-05). All animal use and welfare practices followed the National Institutes of Health (NIH) *Guide for the Care and Use of Laboratory Animals* and guidelines of the International Association for the Study of Pain (IASP). All possible efforts were made to minimize animal suffering during the experiments.

Adult male C57BL/6J mice (8–10 weeks old, 22–28 g) were purchased from Japan SLC, Inc. (Hamamatsu, Japan). Mice were housed and maintained on a 12-h light-dark cycle with sufficient water and food. Animals were bred and maintained in accordance with the current *Guide for the Use of Laboratory Animals* (National Academy Press, Washington DC).

### Drug administration

Lambda carrageenan, NNC 55–3096 and mibefradil were purchased from Sigma-Aldrich (Tokyo, Japan). Capsaicin was obtained from Wako Pure Chemical Industries (Osaka, Japan). Carrageenan, mibefradil and NNC-55-3096 were dissolved in saline, and the pH was adjusted to 7.4. Capsaicin was dissolved in 10% dimethyl sulfoxide (DMSO) at the highest dose used. Carrageenan and capsaicin were administered to the mice at a volume of 50 μL/paw by intraplantar (i.pl.) injection. Capsaicin (10 μg/paw) [21] was administered 2 h or 2 days after carrageenan injection. Mibefradil (65.8 nmol/paw) [7] and NNC 55–3096 (1 nmol/paw) [22] were administered 1 h before carrageenan injection and twice daily thereafter at a volume of 20 μL/paw by i.pl. injection. Control groups received an injection of the corresponding vehicle in all cases.

### Inflammation models

Peripheral inflammation was generated by an i.pl. injection of carrageenan into the right hindpaws of mice. We used 3% carrageenan to induce sub-acute inflammation as described previously [23,24]. Mice were divided into 2 groups. Anesthetized mice received a single injection of saline (50 μL/paw) or carrageenan (50 μL/paw) in the plantar surface of the right hindpaw (Day 0) using a 28-gauge needle (n = 25 saline-injected mice; n = 27 carrageenan-injected mice for RT-PCR, western blotting and immunohistochemical analyses; n = 29 carrageenan-injected mice for the experiments with capsaicin and/or T-type Ca^2+^ channel blockers). Behavioral assessments (von Frey test) were conducted at 10 min, 30 min, 60 min, 1 day, and 2 days after inflammation induction. After behavioral assessments on Day 1 (n = 7 saline-injected mice and n = 7 carrageenan-injected mice) or Day 2 (n = 14 saline-injected mice and n = 14 carrageenan-injected mice), the 5th lumbar (L5) DRGs were harvested for subsequent RT-PCR, qRT—PCR and western blotting analyses.

### Assessment of mechanical hyperalgesia

The mechanical nociception threshold was evaluated using von Frey filaments (Sakai Iryou Co., Tokyo, Japan), as previously described [25]. Briefly, a series of von Frey filaments (0.03, 0.07, 0.17, 0.41, 0.70, 1.19, 1.49, 2.05, 3.63, 5.50, and 8.65 g) were used to assess the thresholds of mechanical stimulation. Mice were placed on an elevated wire mesh platform and left to acclimate for 1 h. Filaments were then applied to each hindpaw with a 30-s interval between each application, and the test was repeated for a total of 5 times. The nociceptive threshold was considered to have been passed when the mouse lifted its paw in response to the von Frey filament more than 3 times.

### Assessment of thermal hyperalgesia

The thermal nociceptive threshold was evaluated using a hot plate as previously described [26]. Briefly, the plantar side of the hindpaw was placed on a hot-plate surface (55 ± 1°C). The latency for hindpaw withdrawal from the hot-plate surface was manually recorded using a chronometer. Two measurements were taken with a 180-s interval between each trial and then averaged. For the behavioral tests ("Assessments of mechanical and thermal hyperalgesia"), blinding and randomization were performed by four investigators who were not involved in these analyses.

### Reverse transcription polymerase chain reaction (RT-PCR)

Mice were decapitated under deep anesthesia with sodium pentobarbital (80 mg/kg i.p.; supplemented as necessary) [n = 15 saline-injected mice and n = 15 carrageenan-injected mice for the RT-PCR and quantitative RT-PCR (qRT-PCR) analyses]. Left and right L5 DRGs were separately collected from mice treated with carrageenan or saline 1 and 2 days after injection and then rapidly frozen and stored at -80°C until further processed. Total RNA of the DRGs was isolated using the Isogen reagent (Wako Pure Chemical Industries, Osaka, Japan) according to the manufacturer’s instructions. Total isolated RNA (3 μg) was mixed with 1 μL of random primers (150 ng) (Invitrogen, Carlsbad, CA, USA) and 5 μL of a 2 mM dNTP mix (Applied Biosystems Japan Ltd., Tokyo, Japan), heated to 65°C for 5 min and incubated on ice for at least 1 min. Then, 4 μL of 5× First-strand Buffer, 1 μL of 0.1 M DTT, 1 μL of RNase inhibitor (Invitrogen) and 1 μL of SuperScript III Reverse Transcriptase (Invitrogen) were added to the mixture and incubated at 25°C for 5 min and 50°C for 1 h for reverse transcription (RT). Next, each sample was amplified through 32 PCR cycles with the following primers: Cav3.1 (GenBank Accession No. NM_009783), sense 5’-GGAGCTGGAGCTAGAGATGA-3’ (6051–6070) and antisense 5’-CAGACAAGATGGAGCCTGACT-3’ (6401–6421) (product = 371 base pairs [bp]); Cav3.2 (NM_021415), sense 5’-TCTCTGAGCCTCTCACGGAT-3’ (6336–6355) and antisense 5’-GATGTGGCTGACCTCCTCAT-3’ (6616–6635) (product = 300 bp); Cav3.3 (NM_001044308), sense 5’-CTGGAGACCTGGATGAATGCT-3’ (6188–6208) and antisense 5’-CAAGAGGGTGCAGTTGACACT-3’ (6493–6513) (product = 326 bp). The resulting PCR products were separated by electrophoresis on a 1% agarose gel. DNA sequencing confirmed the molecular identity and homogeneity of the resulting PCR products.

Isolated total RNA was treated with DNase at 37°C for 5 h and subjected to random-primed reverse transcription using SuperScript III as described above for the qRT-PCR analyses. qRT-PCR was performed using a 7500 Fast Real-Time PCR System (Applied Biosystems) with Power SYBR Green PCR Master mix (Applied Biosystems) and primers specific to each gene. Samples were subjected to 40 cycles of 95°C for 10 s and 60°C for 1 min after preincubation at 95°C for 10 min, followed by a dissociation stage to ensure that all primer pairs only annealed to the desired product. The following sequences of specific primers were used: Cav3.2 sense 5’-GGCTGGGTGGACATCATGTACT-3’ (1467–1488) and antisense 5’-CCACCAGGCACAGGTTGATCAT-3’ (1560–1581) (product = 115 bp) and ß-actin (GenBank Accession No. NM_007393) sense 5’-ACCATGTACCCAGGCATTGC-3’ (989–1008) and antisense 5’-GCTAGGAGCCAGAGCAGTAATCT-3’ (1026–1048) (product = 60 bp). Validity was determined using a dissociation curve with a single peak and standard curve values (slope and R^2^). Average Ct values from triplicate PCR reactions were normalized to the average Ct values of the reference gene (ß-actin) from the same cDNA template. We determined the difference in gene expression by calculating the ratio of Cav3.2/ß-actin relative to the corresponding saline control in the linear range using densitometric analysis with ImageJ software.

### Western blotting analysis

Total protein extracts were obtained from ipsilateral L5 DRGs in mice treated with saline or carrageenan (n = 6 mice for each group). Equal amounts of protein (20 μg) were separated using SDS-PAGE electrophoresis (7.5% for Cav3.2 and 10% for ß-actin). Cav3.2 was detected using a goat anti-Cav3.2 polyclonal antibody targeting a peptide at the N-terminus of the Cav3.2 channel (N-18, sc-16261, Santa Cruz Biotechnology, Santa Cruz, CA, USA), followed by a horseradish peroxidase-labeled donkey anti-goat IgG secondary polyclonal antibody (sc-2022, Santa Cruz Biotechnology). Antibodies were visualized using an ECL Western blotting Detection kit (GE Healthcare, Little Chalfont, Buckinghamshire, UK). We tested three commercial anti-Cav3.2 antibodies for western blot analyses (Santa Cruz Biotechnology N-18, Alomone Lab ACC-025 and Sigma-Aldrich C1868). The specificity of the anti-Cav3.2 antibody from Santa Cruz Biotechnology (N-18) has been confirmed in HEK-293 cells stably expressing the Cav3.2 channel [27] and Cav3.2 knockout mice [28]. In addition, our preliminary study found that this antibody could be used to accurately determine the quantity of Cav3.2 channel proteins in mouse DRG tissues via visualization of an ~250 kDa band (S1 Fig). The level of ß-actin was evaluated using a rabbit anti-actin polyclonal antibody (4967S, Cell Signaling Technology Inc., Danvers, MA, USA) followed by a horseradish peroxidase-labeled anti-rabbit IgG secondary polyclonal antibody (W401B, Promega, Madison, WI, USA), which was then visualized with the ECL detection kit. We determined the difference in protein expression by calculating the ratio of Cav3.2/ß-actin using densitometric analysis using ImageJ software.

### Preparation for histological analyses

Untreated (n = 8), saline- and carrageenan-treated (n = 3 each) mice were transcardially perfused and fixed with 4% paraformaldehyde (PFA) in 0.1 M phosphate-buffered solution (PB, pH 7.4) under anesthesia. The L5 DRGs were excised, postfixed in the same fixative solution and cryoprotected overnight in a phosphate-buffered 30% sucrose solution. The cryosections were prepared for *in situ* hybridization and double-staining immunohistochemistry.

### *In situ* hybridization

A plasmid containing mouse Cav3.2 cDNA (GenBank: NM_021415: nucleotides 6321–6978) was linearized with SalI for antisense RNA synthesis by T3 polymerase; XbaI linearized the cDNA for sense RNA synthesis by T7 polymerase (Promega). Probes were labeled with [^35^S]-dUTP (PerkinElmer) or digoxigenin (DIG) (DIG RNA labeling mix, 11 277 073 910; Roche Diagnostics GmbH, Mannheim, Germany). *In situ* hybridization techniques were performed according to a method described previously with some modifications [29,30]. For the method using radioisotope-labeled probes (n = 3), PFA-fixed cryosections of mouse DRGs were treated with proteinase K for 10 min at room temperature and then fixed in 4% PFA for 5 min. After being washed in PB, the sections were acetylated, dehydrated and air dried. [^35^S]-labeled RNA probes were denatured and hybridized to the sections for 16 h at 55°C. Slides were subjected to high-stringency washing, treated with RNase A, dehydrated and covered with a photographic emulsion (NTB-2; Kodak). After a 20-day exposure, the sections were developed in a Kodak D-19 developer and fixed with a photographic fixer. For use with the digoxigenin (DIG)-labeled probe, mouse DRG sections (n = 3) were subjected to prehybridization treatments as described above. DIG-labeled probes for Cav3.2 mRNA were denatured and hybridized for 20 h at 55°C. Slides were subjected to high-stringency washing and incubated with an alkaline phosphatase-conjugated goat anti-DIG Fab fragment (1:500) (11 093274 910, Roche Diagnostics). Signals were detected using nitroblue tetrazolium chloride (NBT) (Roche Diagnostics) and 5-bromo-4-chloro-3-indolyl-phosphate (BCIP) (Roche Diagnostics). The sense cRNA probe was used to confirm the specificity of the hybridization signals obtained using the antisense cRNA probe.

### Double-staining immunohistochemistry

PFA-fixed cryosections of DRGs were obtained from mice sacrificed 2 days after carrageenan or saline injection (n = 3 each); the cryosections were prepared at a thickness of 20 μm and mounted on MAS-coated glass slides (Matsunami Glass Ind., Ltd., Osaka, Japan). Sections were air dried and blocked with 5% normal donkey serum in phosphate-buffered saline (PBS) containing 0.3% Triton X-100 (blocking buffer) for 30 min at room temperature and then incubated with a rabbit anti-Cav3.2 polyclonal antibody against CHVEGPQERARVAHS, which corresponds to amino acid residues 581–595 of rat Cav3.2 (1:500, C-1868, Sigma-Aldrich, Tokyo, Japan) [31], and either a goat anti-transient receptor potential vanilloid 1 (TRPV1) polyclonal antibody (1:500, P-19, sc-12498, Santa Cruz Biotechnology), goat anti-peripherin polyclonal antibody (1:100, C-19, sc-7604, Santa Cruz Biotechnology), goat anti-neurofilament-H (NF-H) polyclonal antibody (1:200, E-15, sc-22909, Santa Cruz Biotechnology), goat anti-calcitonin gene-related peptide (CGRP) polyclonal antibody (1:300, 1720–9007, AbD Serotec, Kidlington, UK) or Alexa Fluor 488-conjugated isolectin GS-IB4 (1:100, 121411, Invitrogen) in blocking buffer overnight at 4°C. Sections were incubated with an Alexa 594-conjugated donkey anti-rabbit IgG (Invitrogen; 1:1000) and/or an Alexa 488-conjugated donkey anti-goat IgG (Invitrogen; 1:1000) polyclonal antibody for 1 h at room temperature. All immunohistochemical images were acquired using an Olympus AX70-based fluorescence microscope (Olympus Co., Tokyo, Japan), and the immunoreactive cells and total DRG neurons were counted. The specificity of the anti-Cav3.2 antibody was confirmed by signal ablation using an antigenic peptide (S2 Fig).

### Statistical analyses

Pooled data are shown as the mean ± SEM. Differences between groups were analyzed using an unpaired Student’s *t-test*, one-way analysis of variance (ANOVA) and two-way ANOVA, followed by the Bonferroni post hoc test, although a power calculation was not employed. Differences were considered significant at p < 0.05.

## Results

### Development of mechanical hyperalgesia after intraplantar carrageenan injection

**Fig 1:**
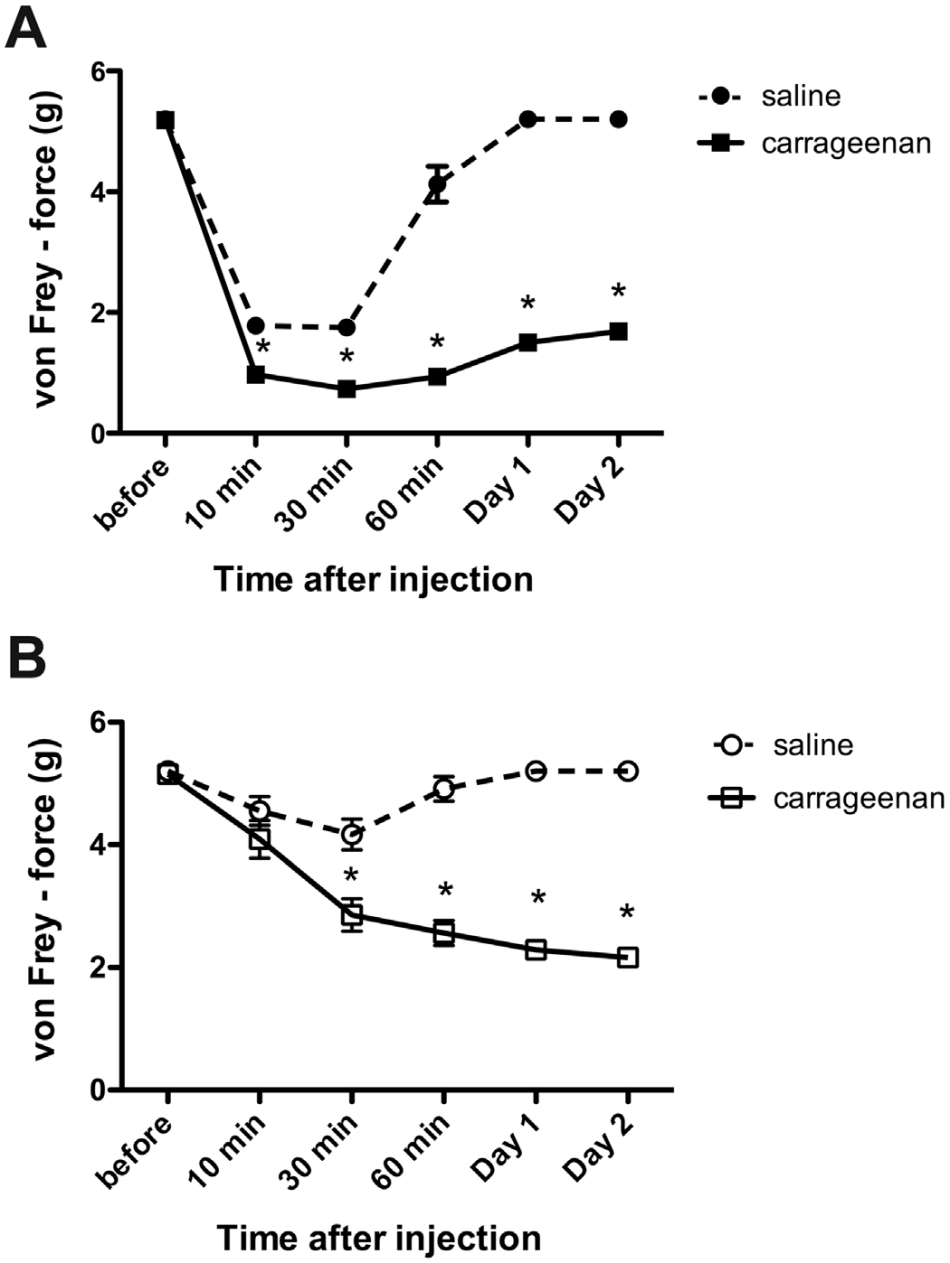
Intraplantar carrageenan injection induces inflammation-mediated mechanical hyperalgesia. Paw withdrawal thresholds (PWT) following saline or carrageenan injection were determined using the von Frey test on the ipsilateral (A) and contralateral sides (B). (n = 25 mice for saline and n = 27 mice for carrageenan). *p < 0.05 compared with the respective saline-treated mice (two-way ANOVA with the Bonferroni post hoc test).

The baseline paw withdrawal threshold (PWT) values in the von Frey test for the right and left hindpaws of untreated mice were 5.34 ± 0.10 g and 5.33 ± 0.11 g, respectively (Fig 1). These values were consistent with previous studies that showed the baseline threshold of mechanical nociception in naïve mice was approximately 5.50 g [25,26]. Subcutaneous carrageenan administration to the right hindpaw caused ipsilateral inflammation of the treated paw with marked swelling and redness in addition to reductions in the mechanonociceptive thresholds (Fig 1). Hypersensitivity was first observed 10 min after the injection and peaked at 30 min. The hypersensitivity recovered slightly but persisted throughout the sub-acute phase (Days 1–2) with a mean PWT threshold of 1.89 ± 0.16 g (Fig 1). Hyperalgesia was also observed on the contralateral side (Fig 1), but there was no marked swelling or redness. In contrast to that on the ipsilateral side, the mechanonociceptive threshold on the contralateral side gradually decreased and plateaued on Day 1. The hyperalgesia observed on Day 2 was similar in magnitude to that on the ipsilateral side, with a mean PWT threshold of 2.17 ± 0.33 g (Fig 1; contra carrageenan). A slight hypersensitivity was observed in the ipsilateral hindpaws of mice injected with saline during the acute phase, but this parameter returned to baseline on Day 1 (Fig 1). Therefore, unilateral carrageenan injections caused mechanical hyperalgesia on the ipsilateral and contralateral sides in our model animals.

### Upregulated expression of Cav3.2 mRNA and protein in DRG neurons during the sub-acute phase of carrageenan-induced hyperalgesia

The T-type current density increases in acutely dissociated, small DRG neurons in rats with diabetic neuropathy [32] and in rats with mechanical injury of the spinal nerve [15]. Yue and coworkers suggested that enhanced T-type currents during nerve injury are due to upregulated gene expression of Cav3.2 channels [15], but they did not examine protein expression. In contrast, Takahashi et al. reported that Cav3.2 protein levels were dramatically upregulated after the L5 spinal nerve was cut in rats [33], but they did not examine whether the upregulated protein expression resulted from enhanced gene expression. We reasoned that the enhanced activities of T-type calcium channels during pathological pain are due to qualitative changes, including the sensitization of Cav3.2 channels, and quantitative alterations that involve upregulation at the gene and protein levels.

**Fig 2:**
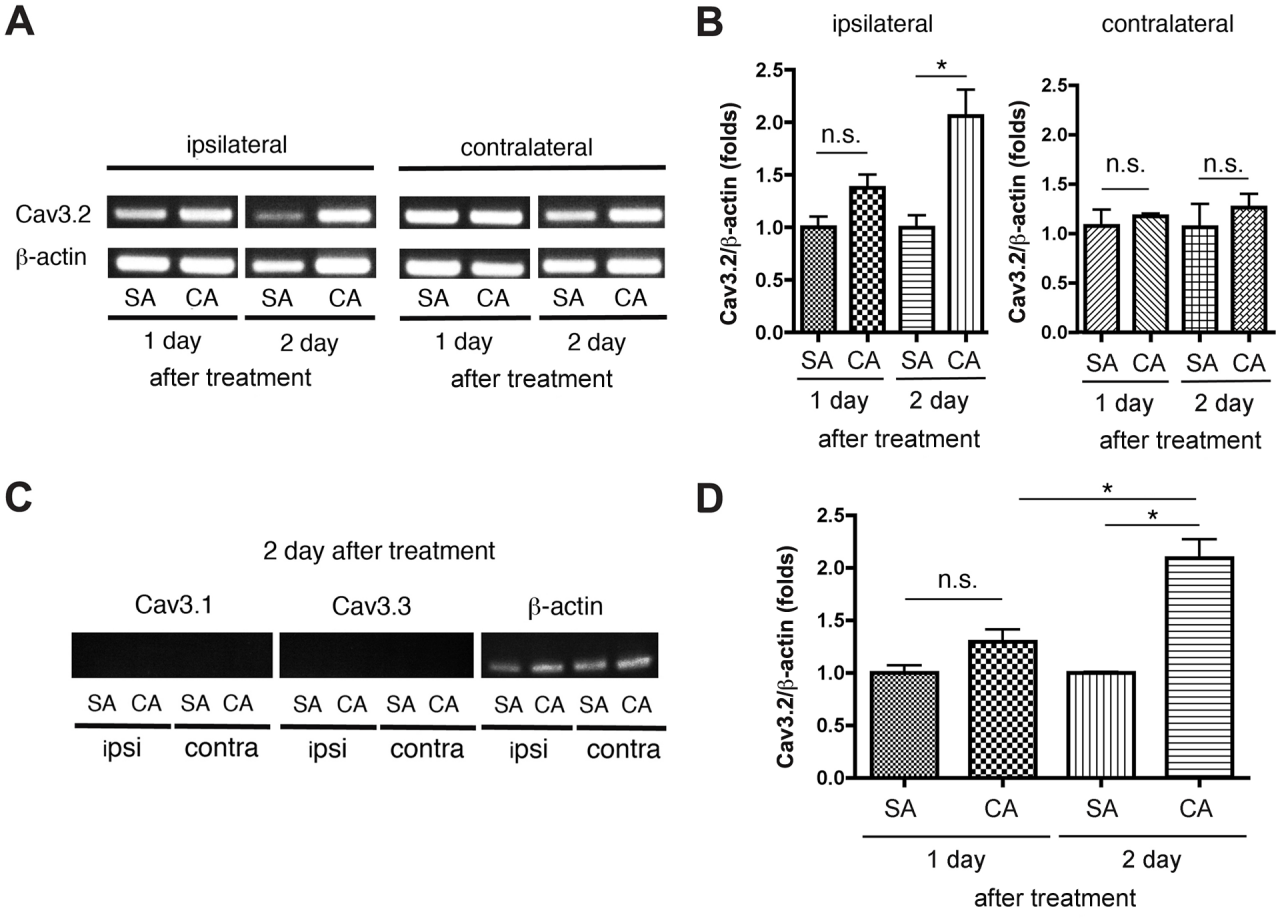
Cav3.2 mRNA is gradually upregulated in ipsilateral L5 DRG tissues during the sub-acute phase (Days 1–2) following carrageenan injection. (A and B) Representative gel images of semi-quantitative RT-PCR analyses showing an upregulation of Cav3.2 mRNA (A) and data (proportion relative to ß-actin) quantified by densitometry (B). (C) Representative images of semi-quantitative RT-PCR analyses of Cav3.1 and Cav3.3 mRNA on Day 2 showing no upregulation (n = 3 mice for Day 1 and n = 4 mice for Day 2). (D) Summary of data from the quantitative RT-PCR (qRT-PCR) analyses. The results were similar to the results of the semi-quantitative RT-PCR: Cav3.2 mRNA was increasingly upregulated during the sub-acute phase of hyperalgesia (n = 4 mice for each group). *p < 0.05 compared with saline-treated controls (one-way ANOVA with the Bonferroni post hoc test). n.s., not significant; SA, saline; CA, carrageenan; ipsi, ipsilateral; contra, contralateral.

**Fig 3:**
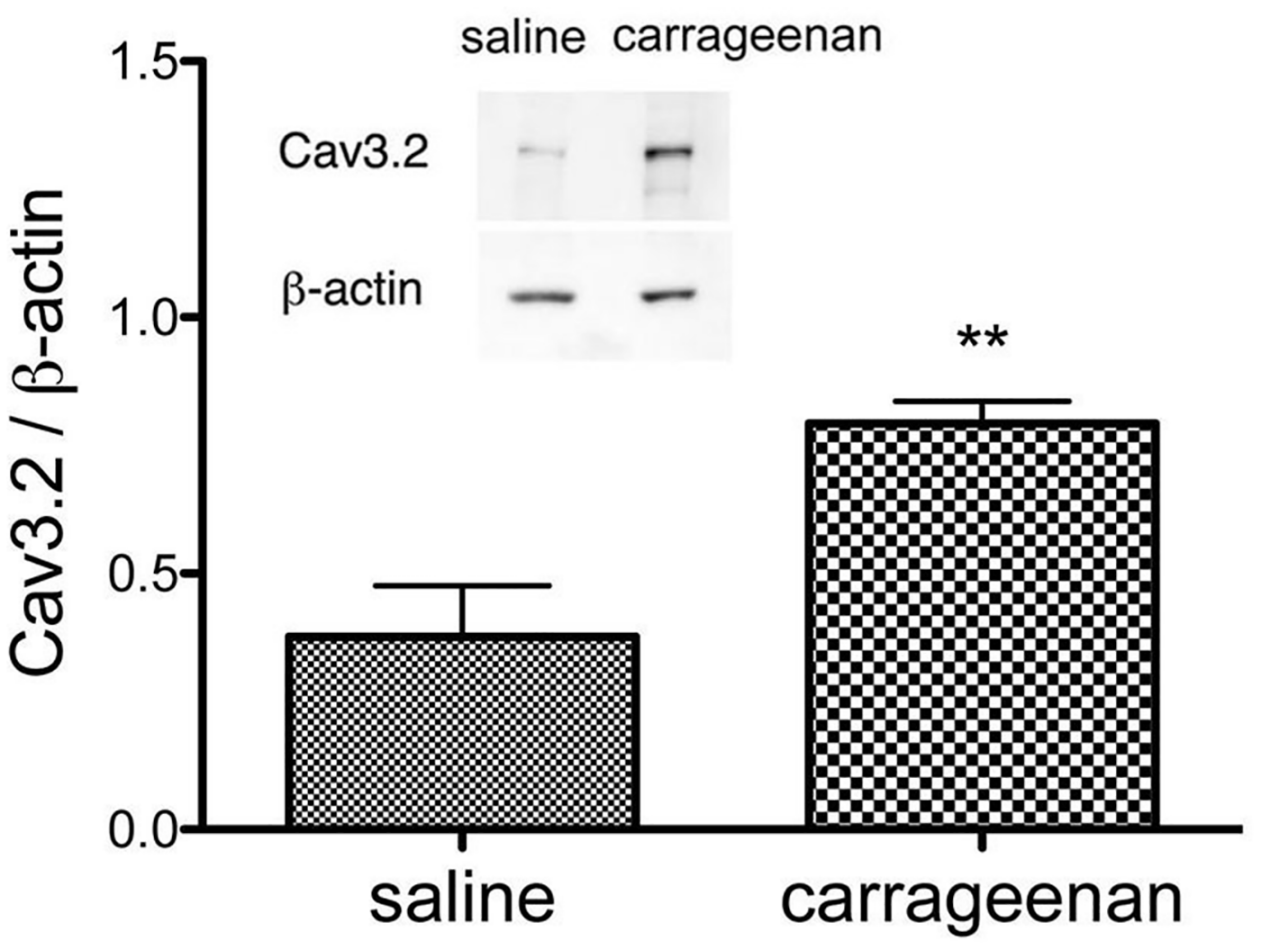
Cav3.2 protein expression is enhanced in ipsilateral L5 DRG tissues during the sub-acute phase (Day 2) following carrageenan injection. Cav3.2 protein expression was analyzed and normalized relative to the expression of the housekeeping protein ß-actin. The inset of a representative, original immunoblot shows the up-regulation of Cav3.2 protein (n = 6 mice for each group). *p < 0.05 compared with the saline control (unpaired Student's *t-test*).

We first performed semi-quantitative RT-PCR analysis using the total RNA obtained from L5 DRGs 1 and 2 days after injection to examine the changes in the expression of T-type Ca^2+^ channel genes in primary afferent neurons during the sub-acute phase of carrageenan-induced inflammatory pain. Fig 2A and 2B show that Cav3.2 mRNA was gradually upregulated in ipsilateral DRGs that projected to the hindpaw treated with carrageenan during the sub-acute phase (Days 1 and 2) compared with that in ipsilateral DRGs treated with saline. In contrast, Cav3.2 mRNA was not altered in contralateral DRGs treated with carrageenan (Fig 2A and 2B), and Cav3.1 and Cav3.3 did not exhibit increased gene expression in the ipsilateral or contralateral DRGs treated with carrageenan (Fig 2C). We also performed qRT-PCR using a SYBR Green dye indicator (Fig 2D) to further confirm the upregulation of Cav3.2 mRNA in DRGs from mice with inflammation-induced hyperalgesia. The qRT-PCR analyses clearly verified that Cav3.2 mRNA was progressively upregulated in the ipsilateral DRGs 1.2-fold on Day 1 and 2.1-fold on Day 2 compared with that in the ipsilateral DRGs treated with saline. Therefore, Cav3.2 mRNA, but not Cav3.1 and Cav3.3 mRNA, was upregulated in ipsilateral DRG neurons projecting to the mouse hindpaw with inflammatory hyperalgesia. We next examined protein expression by western blot analysis. Cav3.2 protein expression increased in ipsilateral L5 DRG neurons during the sub-acute phase (Day 2) in carrageenan-treated mice (Fig 3). These findings suggest that the upregulation of the Cav3.2 gene increased Cav3.2 protein levels, which may be associated with the maintenance of ipsilateral inflammatory hyperalgesia during the sub-acute phase.

### Characterization of Cav3.2 channels in mouse DRG neurons

**Fig 4:**
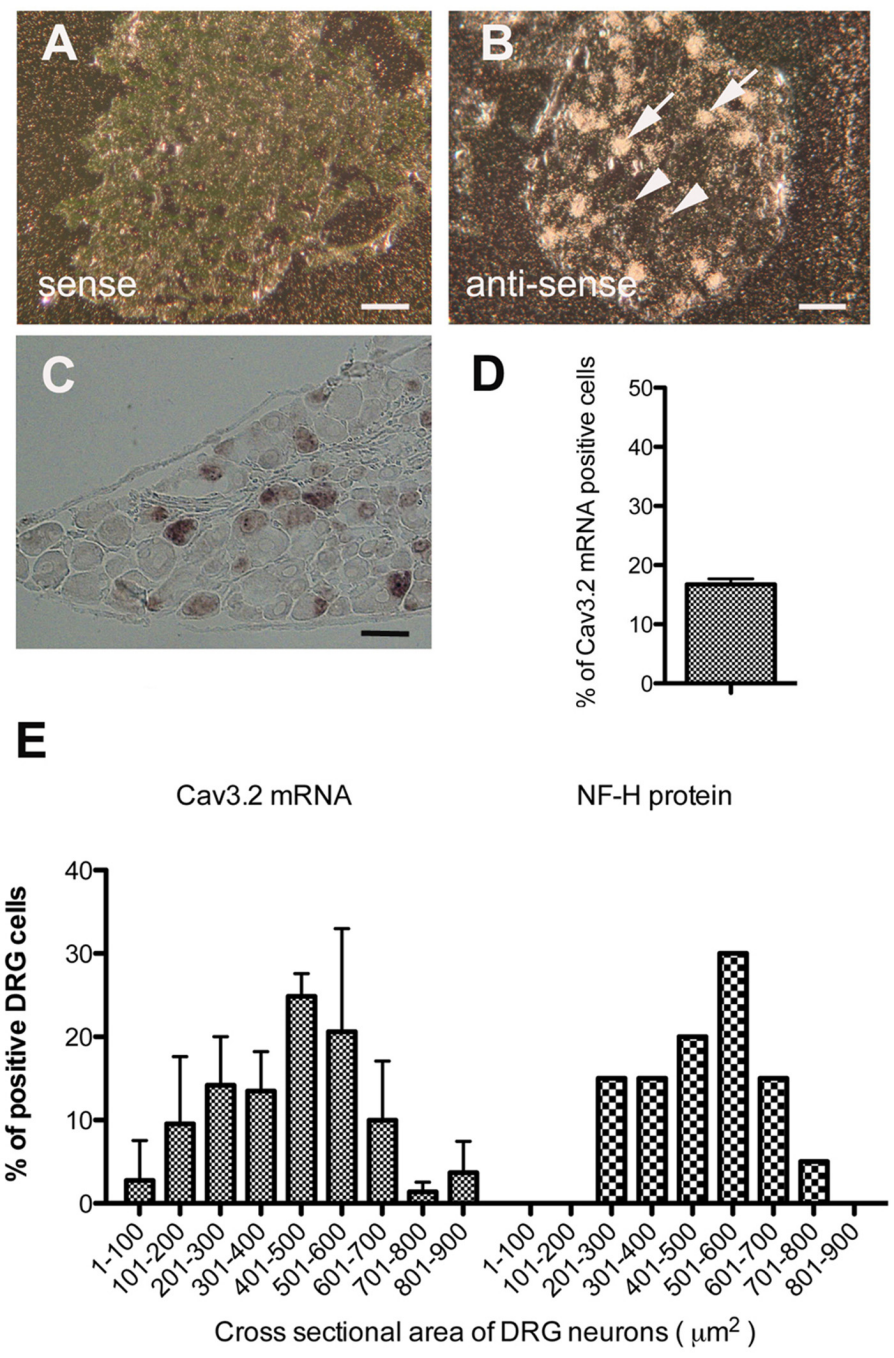
Cav3.2 mRNA is expressed in a subset of DRG neurons in normal mice. (A and B) Macroautoradiographic images of an [^35^S]-labeled probe from an *in situ* hybridization showing the specificity of the Cav3.2 probe (A; sense probe) and Cav3.2 mRNA expression (B; anti-sense probe) in mouse DRG tissues. Hybridization signals were obtained only when the anti-sense probe was used. The medium DRG neurons (arrows) were densely labeled, whereas smaller neurons (arrow head) were only weakly labeled. Scale bar = 50 μm (C) Microscopic images of a DIG-labeled probe for *in situ* hybridization (n = 3 mice for each probe). Scale bar = 50 μm. (D) The proportion of Cav3.2 mRNA-positive neurons relative to the total number of DRG neurons was determined. (E) Histogram showing the proportions of Cav3.2 mRNA-labeled cells based on a cross-sectional area (left). The proportion of cells labeled by an anti-NF-H antibody is shown as a reference on the right side. Only labeled cells that included nuclei were subjected to area measurements.

We first examined the expression and localization of Cav3.2 in DRG neurons from untreated normal mice. *In situ* hybridization using different probes ([^35^S]- and DIG-labeled probes) demonstrated that Cav3.2 mRNA was expressed in a subset of DRG neurons. [^35^S]-labeled probes labeled approximately 18% of all DRG neurons (Fig 4B), and DIG-labeled probes labeled approximately 17% of all DRG neurons (Fig 4C and 4D). *In situ* hybridization using [^35^S]-labeled probes also revealed that medium-sized neurons tended to express relatively high levels of Cav3.2 mRNA, whereas smaller neurons contained lower amounts of mRNA than that of larger neurons (Fig 4B). The sizes of most positive DRG neurons (69 ± 8%) ranged from 300 to 700 μm^2^ in cross-sectional area and 20 to 30 μm in estimated diameter (Fig 4E), which indicates that Cav3.2 mRNA was mainly expressed in small and medium-sized DRG neurons in normal mice.

We also examined the expression and localization of Cav3.2 at the protein level. Recently, Rose et al. reported the use of a reliable anti-Cav3.2 antibody (Sigma-Aldrich, C1868), which specifically labeled HEK293 cells expressing the human Cav3.2 isoform but did not exhibit positive reactions in cultured DRG neurons from Cav3.2-knockout mice [31]. We used this antibody to characterize DRG neurons expressing Cav3.2 channel proteins in mice treated with saline or carrageenan. Cav3.2 immunostaining in saline-treated mice was observed in a subset of L5 DRG neurons. The percentage of Cav3.2-positive cells was 22 ± 3% of the total cells on the ipsilateral side and 19 ± 2% of the total cells on the contralateral side; these values are similar to the percentages obtained in prior *in situ* hybridization studies of Cav3.2 mRNA. Furthermore, sections were double-labeled with Cav3.2 and an established sensory neuronal marker, such as peripherin, IB4, CGRP, TRPV1 or NF-H. Approximately 56% of all Cav3.2-immunoreactive (IR) DRG neurons were co-labeled with NF-H, which labels myelinated fibers, and 46% were co-labeled with peripherin, which labels thin, unmyelinated fibers. Co-immunolocalization with IB4 and CGRP was found in approximately 34% and 37% of Cav3.2-IR DRG cells, respectively. Surprisingly, only 15% of the Cav3.2-IR DRG neurons were co-labeled with TRPV1, but only 10% of the TRPV1-positive cells showed Cav3.2 immunostaining. Therefore, Cav3.2 channels could be abundant in neurons with myelinated fibers and expressed in those with unmyelinated fibers. These channels may have little association with capsaicin- or TRPV1-mediated cellular responses in normal states.

### Alterations in Cav3.2 expression in DRG neurons after carrageenan treatment

**Fig 5:**
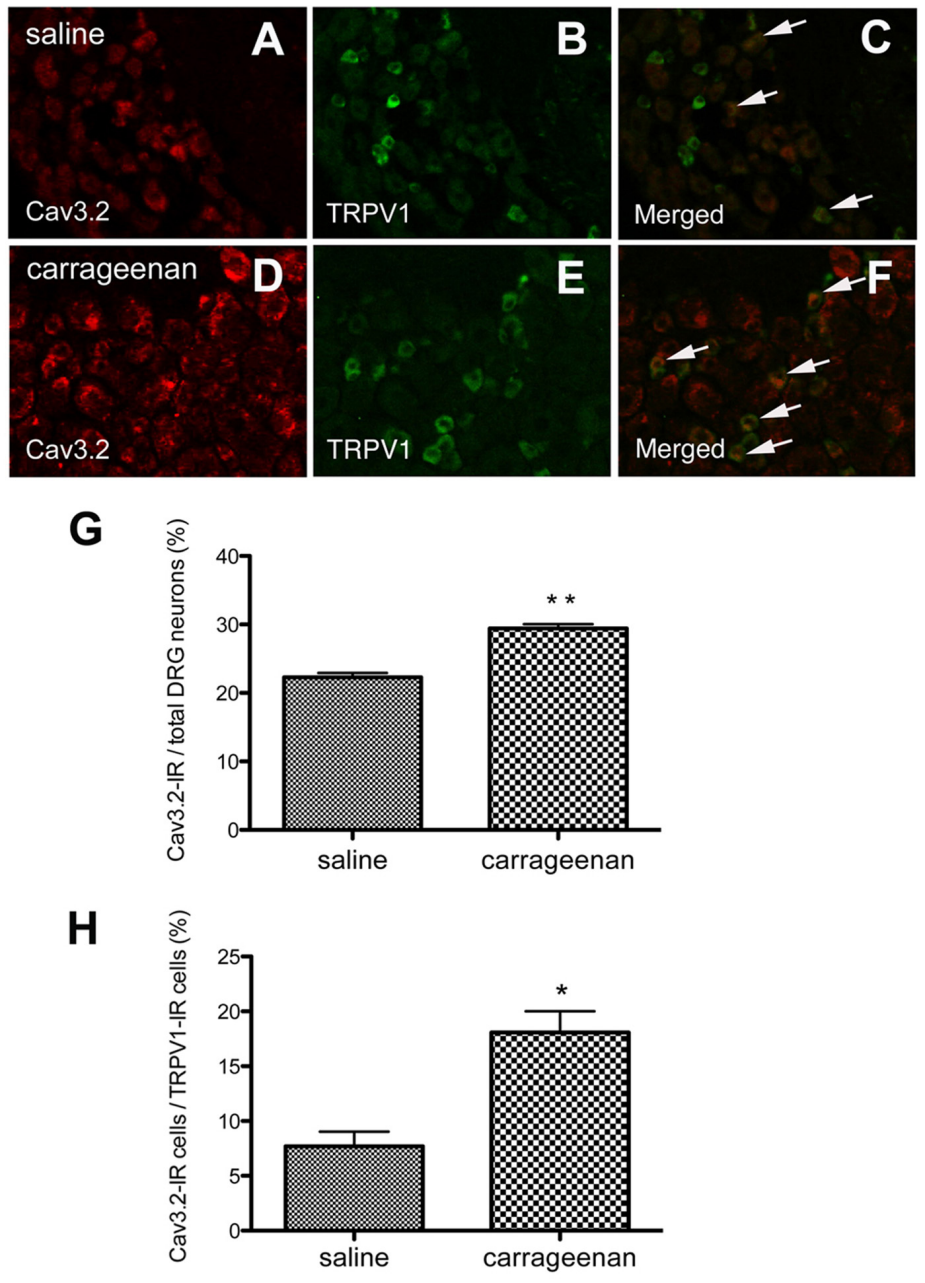
Intraplantar carrageenan injection increases Cav3.2 immunoreactivity in a subpopulation of ipsilateral L5 DRG neurons on Day 2 post-injection. (A-C) In saline-treated mice, representative immunostaining images showing Cav3.2 (A), TRPV1 (B), and both (C). Arrows indicate co-localization of Cav3.2-IR with TRPV1-IR. (D-F) In carrageenan-treated mice, representative immunostaining images showing Cav3.2 (D), TRPV1 (E), and both (F). Arrows indicate co-localization of Cav3.2-IR with TRPV1-IR. (G) The percentage of Cav3.2-immunoreactive cells relative to all L5 DRG neurons from mice treated with saline or carrageenan. (H) The percentage of Cav3.2-immunoreactive cells in TRPV1-positive neurons from mice treated with saline or carrageenan. (n = 3 mice for each group). *p < 0.05 compared with the saline control (unpaired Student's *t-test*).

We immunostained the ipsilateral L5 DRGs of mice 2 days after carrageenan treatment and measured the percentages of Cav3.2-IR neurons and the co-expression of their markers in carrageenan-injected mice in comparison with saline-treated mice. We found that the Cav3.2-IR neurons in ipsilateral DRG neurons increased 1.5-fold in mice treated with carrageenan (Fig 5A, 5D and 5G). Furthermore, the Cav3.2 immunostaining tended to increase in all of the DRG neurons that were positive for the examined markers (S3 Fig), and it was significantly enhanced in the TRPV1-positive DRG neurons (Fig 5C, 5F, 5H and S3 Fig). Carrageenan treatment did not significantly increase the percentage of marker-expressing cells relative to the total number of DRG neurons in our model (S4 Fig). These results suggested that Cav3.2 was upregulated in a variety of cell types in DRG neurons, especially TRPV1-IR neurons during the sub-acute phase (Day 2) of inflammatory hyperalgesia.

**Fig 6:**
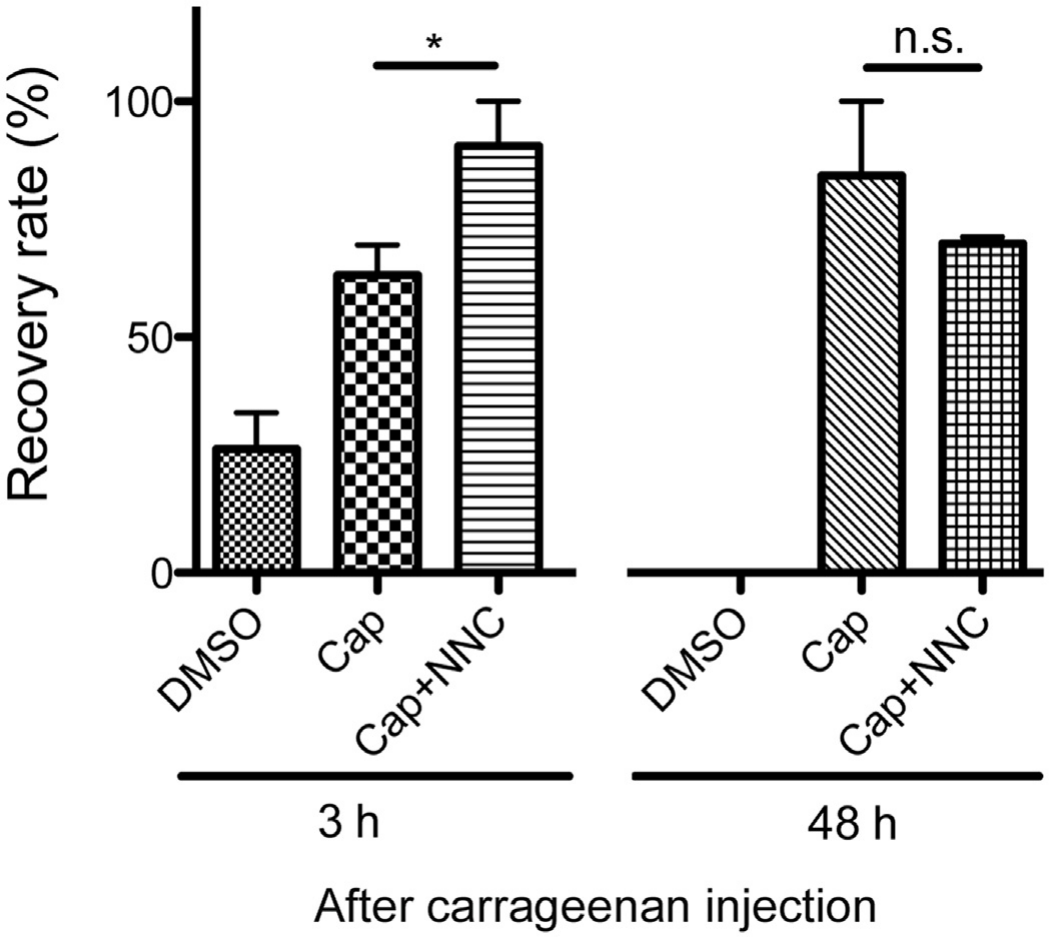
Recovery rates from hyperalgesia after intraplantar injections of capsaicin (cap) or capsaicin and NNC 55–0396 (cap+NNC). At 2 h or 2 days following carrageenan injection, DMSO (control), cap or cap+NNC was injected, and PWTs were evaluated after 1 h, 3 h or 48 h. The data are expressed as relative values, with the recovery rate to baseline of the individual being 100% (n = 3–5 mice for each group). *p < 0.05 compared with capsaicin-treated mice (unpaired Student's *t-test*). n.s., not significant.

The present finding of lower Cav3.2 expression in TRPV1-positive neurons is inconsistent with previous reports that used acutely dissociated neurons from rats [14]. We thus examined the effect of NNC 55–0396, a Cav3.2 channel blocker, on capsaicin-induced analgesia to resolve this discrepancy. Capsaicin and TRPV1 agonists inhibit T-type calcium channels (Cav3.1, Cav3.2 and Cav3.3) through TRPV1 channels in DRG neurons [34,35]. If TRPV1 and Cav3.2 are less co-expressed in DRG neurons during the acute-phase and if their co-expression is increased during the sub-acute phase, then NNC 55–0396 treatment should result in an additional analgesic effect during the acute phase but not the sub-acute phase. In other words, the analgesic effect induced by capsaicin should be enhanced during the sub-acute phase of the inflammatory pain model. We injected capsaicin (10 μg/paw) with or without NNC 55–0396 into the plantar surface of the ipsilateral hindpaw 2 h or 48 h after carrageenan treatment and assessed the effects of these blockers on mechanical hyperalgesia using the von Frey test 1 h after the injection of capsaicin and/or the T-type channel blocker. The analgesic effect of capsaicin tended to increase during the sub-acute phase (48 h) compared with the acute phase (3 h) (Fig 6). In summary, simultaneous administration of NNC 55–0396 and capsaicin was more effective than capsaicin alone on acute inflammatory hyperalgesia, but the T-type channel blocker did not produce any additional effect during the sub-acute phase (Fig 6).

### The effect of T-type Ca^2+^ channel blockers on the development of hyperalgesia during carrageenan-induced inflammation

**Fig 7:**
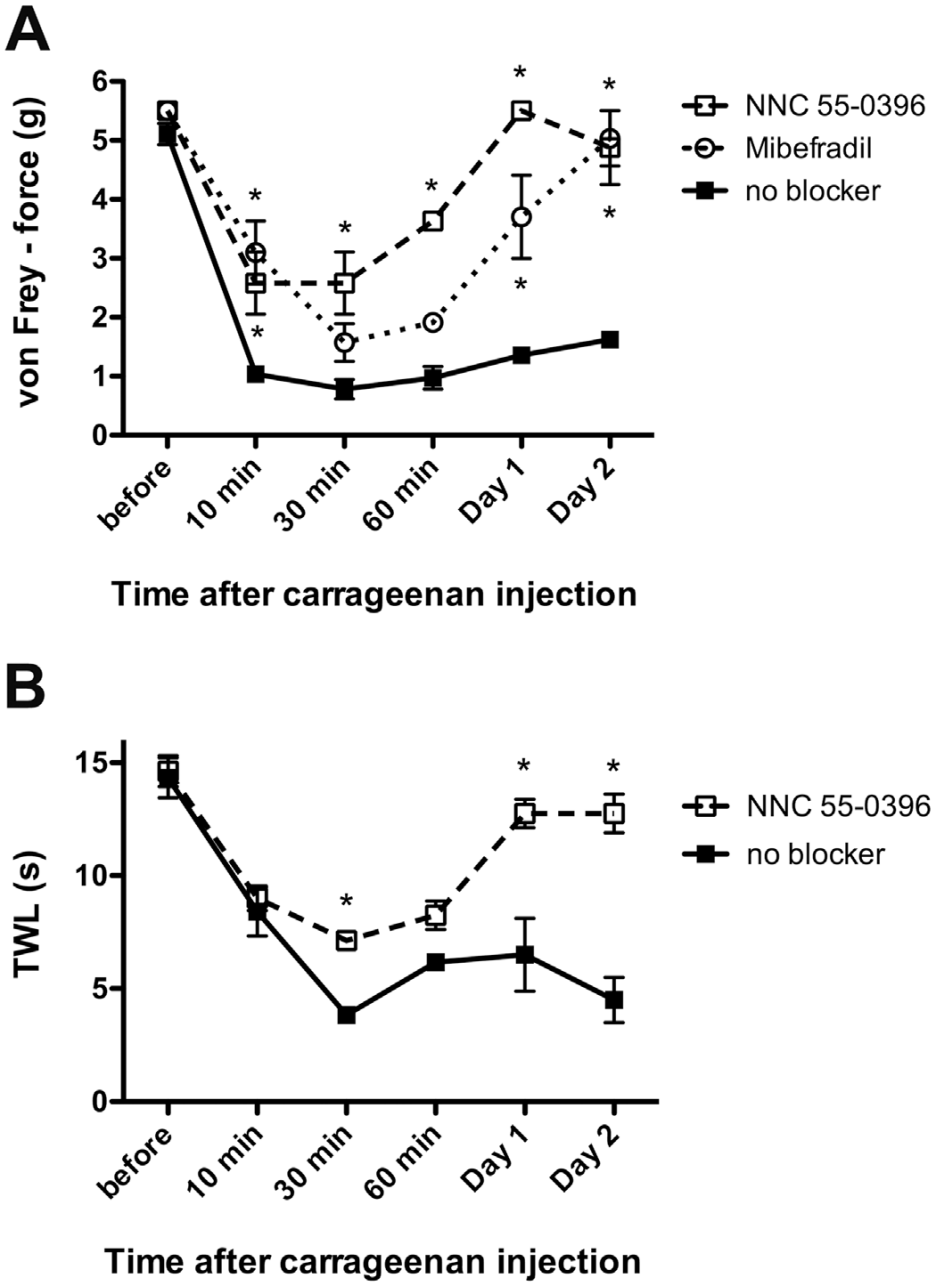
(A) The T-type calcium channel blockers NNC 55–0396 and mibefradil inhibit inflammatory mechanical hyperalgesia. Threshold mechanical force for paw withdrawal (PWT) values during preceding and periodic intraplantar treatments with or without the blocker are indicated. (n = 3 mice for NNC 55–0396, n = 4 mice for mibefradil and n = 19 mice for no blocker). (B) NNC 55–0396 also inhibits inflammatory thermal hyperalgesia. The thermal withdrawal latencies (TWLs) from the hot-plate test during preceding and periodic intraplantar treatments with or without the blocker are indicated (n = 3 mice for NNC 55–0396 and n = 3 mice for no blocker). *p < 0.05 compared with the respective control (no blocker) (two-way ANOVA with the Bonferroni post hoc test). n.s., not significant.

We found that subcutaneous carrageenan injection into the hindpaw caused hyperalgesia on the ipsilateral and contralateral sides. Unilateral carrageenan injection also caused an ipsilateral increase in Cav3.2 protein expression in DRG neurons. These results suggested that the upregulated Cav3.2 protein expression in primary afferent neurons is important in the development of ipsilateral hyperalgesia during inflammatory pain. We determined whether the T-type Ca^2+^ channel blockers mibefradil and NNC 55–0396 could reverse carrageenan-induced hyperalgesia in mice. Mibefradil has strong cardiovascular effects, but a previous study reported that a local mibefradil injection (300 μg) did not affect the systolic or diastolic blood pressure in rats [7]. Our preliminary experiment showed that mibefradil and NNC 55–0396 (single injection before carrageenan treatment) were not effective for more than 12 h after the treatment. Therefore, mibefradil or NNC 55–0396 was initially administered to the right hindpaw before carrageenan treatment and then injected in the same region twice daily. The effects of these drugs at 0 min, 10 min, 30 min, 60 min, Day 1 and Day 2 were evaluated using the von Frey test. Intraplantar mibefradil or NNC 55–0396 treatment alleviated the mechanical hyperalgesia induced by carrageenan treatment, and this treatment was effective during the acute (10–60 min) and sub-acute phases (Days 1–2). The nocifensive behaviors on Day 2 had almost recovered to baseline levels in the ipsilateral hindpaws of mice treated with mibefradil and NNC 55–0396 compared with that in the mice that were not treated with these blockers (Fig 7A). We also examined the effect of NNC 55–0396 on the thermal hyperalgesia induced by intraplantar carrageenan injection, and we found that the T-type calcium channel blocker significantly reduced thermal hypersensitivity during the sub-acute phase (Fig 7B).

## Discussion

T-type Cav3.2 channels modulate the function of peripheral sensory pathways by influencing the excitability of DRG neurons [19]. There is clear evidence that a small subpopulation of sensory neurons in rats and mice express high levels of Cav3.2 mRNA [12–14,36], but the cellular properties conferred by these channels are still debated. We precisely quantified Cav3.2 channels in adult mouse DRGs using a variety of histological experiments and showed that approximately 20% of DRG neurons expressed Cav3.2 mRNA and protein during normal states. T-type channel expression was mainly observed in small and medium-sized neurons ranging from 20 to 30 μm in estimated diameter (Fig 4E). Notably, the medium-sized neurons tended to express relatively high levels of Cav3.2 mRNA, but smaller neurons contained lower amounts of mRNA than that of larger neurons (Fig 4B). These results could reflect previous findings showing that the expression of Cav3.2 can entirely account for the prominent T-type current found in a sub-population of medium-sized sensory neurons [11–13] and that a subpopulation of dissociated DRG neurons in rats (soma size of 26–31 μm), which are referred to as T-rich cells, expressed robust T-type-currents [14]. In contrast, Cav3.2 mRNA was barely detected in larger neurons (> 35 μm in estimated diameter); this finding is consistent with previous electrophysiological studies showing that T-type currents are expressed only in rat DRG neurons that are smaller than 40 μm in diameter [37,38]. Moreover, Cav3.2-immunopositive cells were co-stained with an anti-NF-H antibody, a marker of myelinated A-fibers, and an anti-peripherin antibody, a marker of unmyelinated C-fibers, indicating that Cav3.2 was expressed in both myelinated and unmyelinated neurons. The DRG neurons that were immunoreactive to Cav3.2 were also equally labeled with IB4 and CGRP, markers of non-peptidergic and peptidergic nociceptive neurons, respectively. The present histochemical data demonstrated that 16% of all IB4-positive neurons and 21% of all CGRP-positive neurons co-stained with an anti-Cav3.2 antibody, in accordance with the findings of a study of dissociated DRG neurons from rats (11% of IB4-positive neurons and 19% of CGRP-positive neurons) [31]. Previous studies suggest that non-peptidergic nociceptors showing IB4-positive reactions are associated with the chronic pain induced by nerve injury and that CGRP-positive peptidergic neurons contribute to inflammatory pain [39,40]. Therefore, the present histochemical findings imply that Cav3.2 is involved not only in mechanosensory transduction but also in inflammatory and neuropathic pain.

Previous studies have shown that most acutely dissociated small rat DRG cells (< 31 μm in diameter) that express T-currents are sensitive to capsaicin [4, 14, 41, 42]. Capsaicin is a selective agonist of TRPV1 channels in DRG neurons [43]. Therefore, we asked whether TRPV1-positive neurons co-stained with Cav3.2. Approximately 33% of DRG neurons were labeled with an anti-TRPV1 antibody, which is consistent with the findings of a previous study of mouse DRG neurons (38%) [44]. However, we did not observe a close relationship of Cav3.2 with the capsaicin receptor. We observed Cav3.2 immunostaining in only 10% of the TRPV1-positive neurons in the normal state. Conversely, 15% of Cav3.2-positive neurons co-labeled with TRPV1. We examined the effect of a T-type calcium channel blocker on the analgesic action of capsaicin to resolve this discrepancy. Peripheral application of capsaicin causes persistent desensitization of TRPV1 in nociceptive sensory neurons and produces persistent pain relief [35,45,46]. In addition, a recent study reported that stimulation of TRPV1 inhibits T-type channel currents in rat DRG neurons [35]. If Cav3.2 channels are predominantly expressed in TRPV1-positive neurons, then blockade of T-type calcium channels would not cause any synergic effect with capsaicin-induced analgesia. However, NNC 55–0396, a T-type channel blocker, significantly enhanced the analgesia induced by capsaicin administration 1 h after treatment (Fig 6; 3 h). The sensitivity of the detection of immunofluorescence in fixed neurons differs from that for the functional responses in live neurons [47], but our findings suggest that Cav3.2-expressing neurons are distinct from TRPV1-positive DRG neurons during normal states in the mouse.

Recent reviews have stated that T-type Cav3.2 channels are involved in the processing of pain signals [48] and the development of neuropathic pain, including chronic constrictive injury (CCI), spinal nerve injury (SNI) and diabetic neuropathy [19,20]. Our present finding that Cav3.2 channels are expressed in a subpopulation of CGRP-positive neurons suggests that T-type channels may also contribute to inflammatory pain. To examine this hypothesis, we used an experimental model of inflammatory pain induced by the injection of carrageenan into the mouse hindpaw. The administration of an excessive volume (50 μL) has been used in studies of mechanical hyperalgesia [23]. The possibility that the injection volume could affect hyperalgesic action could be excluded because no prolonged hyperalgesia was observed in the mice injected with same volume of saline, although a slight hypersensitivity was observed immediately after carrageenan treatment (Fig 1). The present study showed that the Cav3.2 mRNA expression was upregulated on Days 1 and 2 during the sub-acute phase of carrageenan-induced inflammatory hyperalgesia. Similarly, Cav3.2 protein expression also increased in ipsilateral DRG neurons during the sub-acute phase. Finally, subcutaneous injection of a T-type channel blocker, mibefradil or NNC 55–0396, before and after carrageenan treatment significantly inhibited the carrageenan-induced inflammatory hyperalgesia in the ipsilateral hindpaw. Interestingly, the enhancement of Cav3.2 immunoreactivity was at least partially due to the increased number of Cav3.2-immunopositive DRG neurons. This finding suggests that carrageenan-induced inflammation changes the cellular properties of certain DRG neurons. Cav3.2 expression tended to increase in all types of DRG cells (peripherin-, NF-H-, IB4- and CGRP-positive neurons) (S3 Fig), but a significant two-fold enhancement in the expression was found in TRPV1-positive neurons. Previously, TRPV1 channels were not believed to be directly sensitive to mechanical stimuli because TRPV1-deficient mice did not exhibit a reduction in mechanical hyperalgesia [49]. However, TRPV1-deficient mice exhibit reduced sensitivity to stretch in colonic afferents [50]. Selective TRPV1 antagonists reduce thermal and mechanical hyperalgesia [51–53]. In addition, a peptide from A-kinase anchoring protein 79 (AKAP79), which blocks TRPV1 sensitization, also inhibits *in vivo* inflammatory thermal and mechanical hyperalgesia [54]. Therefore, these results suggest that the variations in TRPV1-positive neurons caused by persistent tissue inflammation are linked to the development and maintenance of mechanical hyperalgesia and that upregulated Cav3.2 expression may be involved in this process.

The final question was whether T-type channel blockers prevented inflammatory mechanical hyperalgesia. We focused on the role of Cav3.2 in peripheral sensory neurons. Therefore, the blockers, mibefradil and NNC 55–0396, were administered into the right hindpaw. Mibefradil is known to be a classical T-type channel blocker, but it also inhibits L-type calcium channels at higher concentrations via a mechanism that involves intracellular hydrolysis and produces an active metabolite [55]. In contrast, NNC 55–0396, which is synthesized from mibefradil, does not produce the metabolite that causes L-type calcium channel inhibition, making it more selective for T-type calcium channels [56]. Mibefradil or NNC 55–0396 was initially administered into the right hindpaw before carrageenan treatment to examine the role of Cav3.2 in the development of inflammatory hyperalgesia. In addition, these drugs were injected into the same region twice daily because a single injection of these blockers before carrageenan treatment was not effective for more than 12 h after treatment. Preceding and periodic treatments of mibefradil and NNC 55–0396 significantly suppressed carrageenan-induced inflammatory hyperalgesia in the ipsilateral hindpaw; this finding is similar to previous reports on neuropathic pain [15]. Therefore, Cav3.2 channels could play important roles in the development of persistent inflammatory pain.

## Conclusion

We describe a novel finding that Cav3.2 T-type calcium channels are involved in the development and maintenance of the inflammatory hyperalgesia induced by subcutaneous carrageenan injections. Cav3.2 expression was significantly upregulated in TRPV1-positive neurons during the sub-acute phase. Our findings are in line with the hypothesis that an upregulation of T-type channels may increase the excitability of the capsaicin-positive DRG neurons, and these cells may contribute to pathological pain responses, such as mechanical and thermal hyperalgesia [32]. Moreover, treatment with more selective T-type channel blockers effectively alleviated the inflammatory hyperalgesia, which suggests that future pharmacological developments selectively targeted to Cav3.2 T-type channels in primary sensory neurons may offer improved therapy for inflammation-induced hyperalgesia and intractable neuropathic pain.

## Supporting Information

S1 Fig Representative immunoblot images showing the detection of Cav3.2 in mouse DRG tissues. Different amounts of DRG protein samples (10, 20 and 30 μg) were resolved, transferred and immunostained with an anti-Cav3.2 antibody, as indicated. The antibody from Santa Cruz Biotechnology (N-18) specifically detected Cav3.2 channel proteins as an ~250 kDa band.
(TIF)

S2 Fig (A) Cav3.2 immunoreactivity in the dorsal root ganglion (DRG). The Sigma antibody revealed intense positive signals in the cytoplasm of a subset of DRG neurons (arrows). (B) Cav3.2 immunoreactivity in the DRG after pre-absorption treatment with the peptide antigen (CHVEGPQERARVAHS) (10^–6^ M). Scale bars = 30 μm.
(TIF)

S3 Fig Proportion of Cav3.2-immunoreactive (IR) neurons among ipsilateral L5 DRG neurons on Day 2 after treatment with saline or carrageenan. (n = 3 mice for each group). Although carrageenan injection tended to increase the proportion of Cav3.2-immunopositive cells for all markers examined, the proportion of Cav3.2-IR cells significantly increased among the TRPV1-positive DRG neurons. *p < 0.05 compared with the saline-treated control (unpaired Student's *t-test*).
(TIF)

S4 Fig Proportion of neurons showing immunoreactivity to the indicated marker among all ipsilateral L5 DRG neurons on Day 2 after treatment with saline or carrageenan. (n = 3 mice for each group). We observed no change in the proportion of marker-immunopositive cells among all DRG neurons.
(TIF)
