# Supplementary figures and images for: Expression and Regulation of Cav3.2 T-Type Calcium Channels during Inflammatory Hyperalgesia in Mouse Dorsal Root Ganglion Neurons
Source: PLoS One. 2015 May 14;10(5):e0127572. doi: 10.1371/journal.pone.0127572 (PMC4431781; doi:10.1371/journal.pone.0127572)

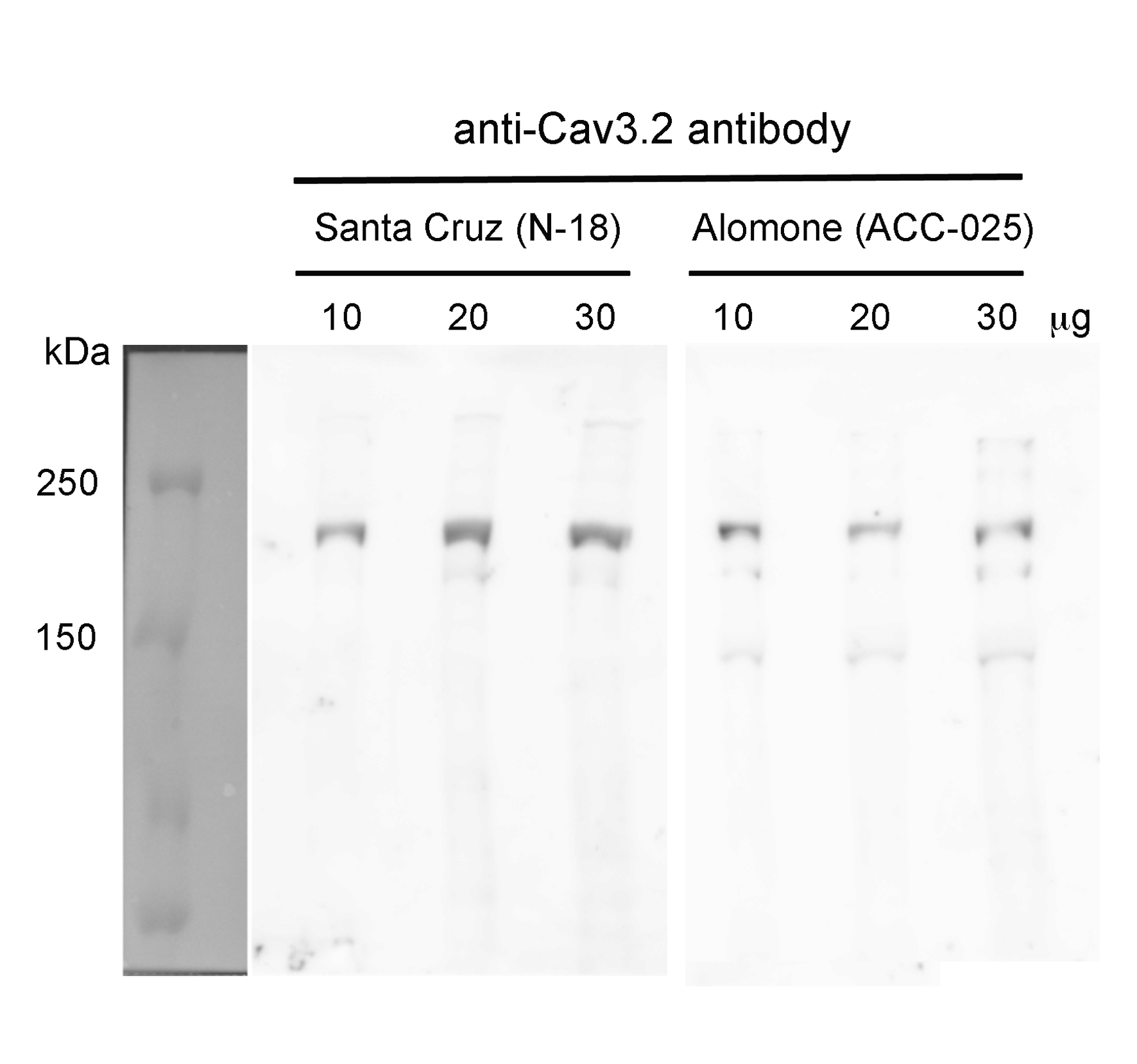

Supplement: S1 Fig — Different amounts of DRG protein samples (10, 20 and 30 μg) were resolved, transferred and immunostained with an anti-Cav3.2 antibody, as indicated. The antibody from Santa Cruz Biotechnology (N-18) specifically detected Cav3.2 channel proteins as an ~250 kDa band. (TIF) [file pone.0127572.s001.tif]

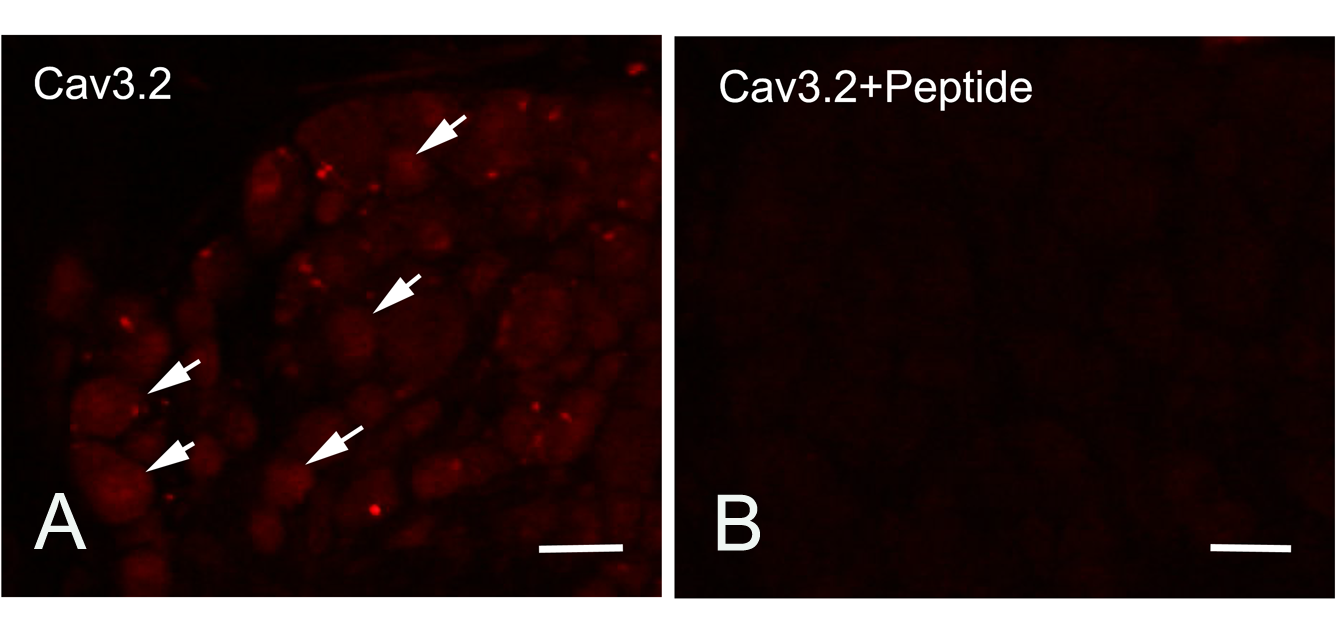

Supplement: S2 Fig — (A) Cav3.2 immunoreactivity in the dorsal root ganglion (DRG). The Sigma antibody revealed intense positive signals in the cytoplasm of a subset of DRG neurons (arrows). (B) Cav3.2 immunoreactivity in the DRG after pre-absorption treatment with the peptide antigen (CHVEGPQERARVAHS) (10–6 M). Scale bars = 30 μm. (TIF) [file pone.0127572.s002.tif]

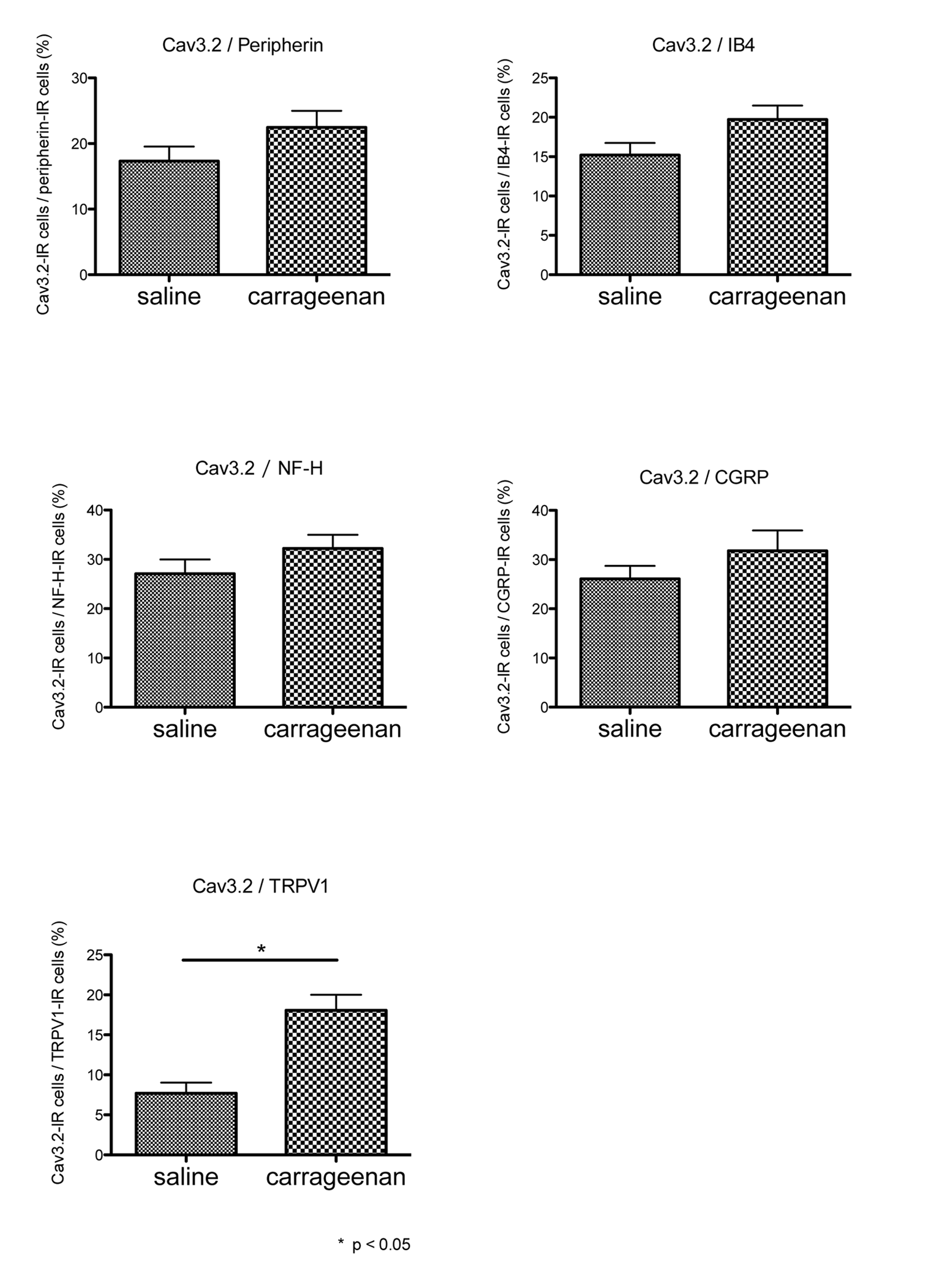

Supplement: S3 Fig — (n = 3 mice for each group). Although carrageenan injection tended to increase the proportion of Cav3.2-immunopositive cells for all markers examined, the proportion of Cav3.2-IR cells significantly increased among the TRPV1-positive DRG neurons. *p < 0.05 compared with the saline-treated control (unpaired Student's t-test). (TIF) [file pone.0127572.s003.tif]

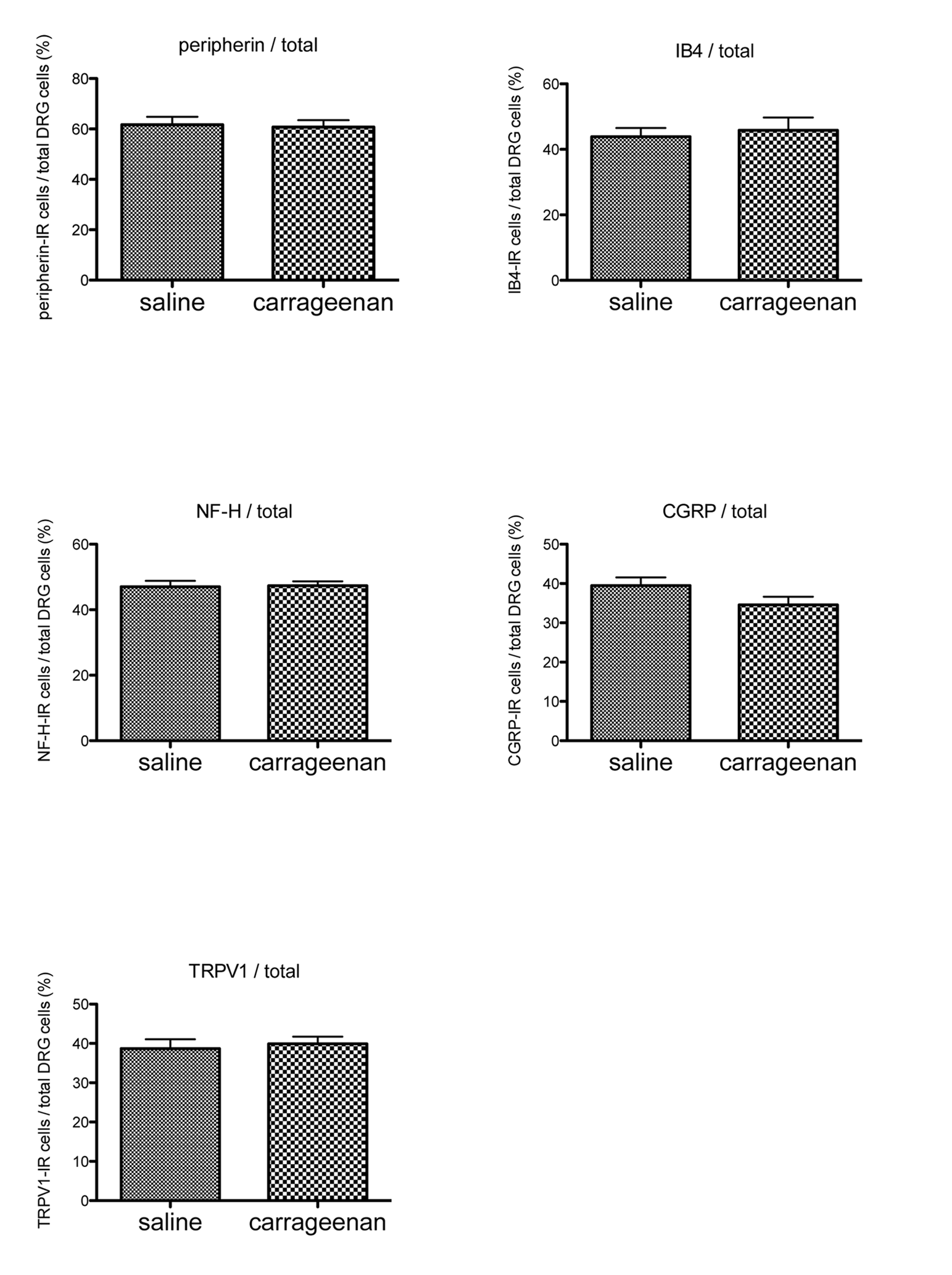

Supplement: S4 Fig — (n = 3 mice for each group). We observed no change in the proportion of marker-immunopositive cells among all DRG neurons. (TIF) [file pone.0127572.s004.tif]
